# Genetic Modulation of Wound Healing Pathways and Postoperative Risk in Plastic and Reconstructive Surgery: A Cohort Study

**DOI:** 10.3390/jcm15072794

**Published:** 2026-04-07

**Authors:** Larysa Sydorchuk, Ruslan Gumennyi, Andrii Sydorchuk, Iryna Batih, Valentina Vasiuk, Ruslan Sydorchuk, Iryna Kamyshna, Pavlo Petakh, Iryna Halabitska, Oleksandr Kamyshnyi

**Affiliations:** 1 Family Medicine Department, Bukovinian State Medical University, Theatre Sq.2, 58002 Chernivtsi, Ukraine; lsydorchuk@ukr.net; 2 Bukovinian Center of Plastic and Aesthetic Surgery, Vladyslav Trepko Str. 1A, 58000 Chernivtsi, Ukraine; plastart.com.ua@gmail.com; 3 Surgery Department, Donauklinik, 89231 Neu Ulm, Germany; andris1595@gmail.com; 4 Department Surgical Dentistry and Maxillofacial Surgery, Bukovinian State Medical University, Theatre Sq.2, 58002 Chernivtsi, Ukraine; batih.iryna@bsmu.edu.ua; 5 Department of Propaedeutics of Internal Diseases, Bukovinian State Medical University, Theatre Sq.2, 58002 Chernivtsi, Ukraine; vasiuk.valentyna@bsmu.edu.ua; 6 General Surgery Department, Bukovinian State Medical University, Theatre Sq.2, 58002 Chernivtsi, Ukraine; rsydorchuk@bsmu.edu.ua; 7 Department of Medical Rehabilitation, I. Horbachevsky Ternopil National Medical University, 46001 Ternopil, Ukraine; kamyshna_ii@tdmu.edu.ua; 8 Department of Biochemistry and Pharmacology, Uzhhorod National University, 88000 Uzhhorod, Ukraine; 9 Department of Therapy and Family Medicine, I. Horbachevsky Ternopil National Medical University, 46001 Ternopil, Ukraine; 10 Department of Microbiology, Virology, and Immunology, I. Horbachevsky Ternopil National Medical University, 46001 Ternopil, Ukraine; kamyshnyi_om@tdmu.edu.ua

**Keywords:** wound healing, transcription factors, plastic surgery, inflammation, mechanism, complications, risk

## Abstract

**Objectives**: The objective of the study was to investigate the mRNA expression of critical gene patterns, including IL-6, CCL2, IL-10, MAPK1, MAPK8, MMP9, COL1A1, COL3A1, and TGFB1, and their associations with adverse postoperative outcomes in reconstructive and plastic surgery patients, depending on age. **Methods**: A total of 95 women participated in this prospective longitudinal cohort study and underwent reconstructive/plastic surgery. The mean age was 35.48 ± 6.61 years (range: 19–57). mRNA expression of IL-6, CCL2, IL-10, MAPK1, MAPK8, MMP9, COL1A1, COL3A1, and TGFB1 genes was evaluated in peripheral blood leukocytes using a PCR-based method with reverse transcription of cDNA. **Results**: The risk of postoperative complications significantly increased with elevated expression levels of IL-6 and COL3A1 (7.5-fold, *p* = 0.007), CCL2 (6.2-fold, *p* = 0.012), and MAPK1 (25.5-fold, *p* < 0.001). Increased expression of MAPK8, IL-10, and MMP9 was associated with a 13.2-fold higher risk (*p* < 0.001). The strongest association was observed for COL1A1 overexpression, which increased complication risk by 58.33-fold (*p* < 0.001). Risk stratification using the Molecular Complication Risk Index (MCRI), incorporating weighted gene contributions, identified an unfavorable molecular profile predominantly among women aged ≥ 40 years. Receiver operating characteristic analysis confirmed the model’s discriminative ability (AUC = 0.78; 95% CI 0.68–0.87), with an optimal cut-off value of MCRI ≥ 8.5 (sensitivity 76%, specificity 71%, *p* < 0.05). **Conclusions**: The transcriptional activity of IL-6, CCL2, IL-10, MAPK1, MAPK8, MMP9, COL1A1, COL3A1, and TGFB1 is associated with postoperative wound healing risk. Women aged over 40 years are at the highest risk of complications. Implementation of the MCRI model may enable early identification of high-risk patients, support targeted preventive strategies, and improve personalized surgical planning.

## 1. Introduction

Wound healing is a fundamental biological process that restores tissue integrity and function following injury. It proceeds through overlapping phases of haemostasis, inflammation, proliferation, and remodelling, each tightly regulated by a complex interplay of cells, cytokines, chemokines, growth factors, and extracellular matrix (ECM) components [1,2,3,4]. Disruption of these processes can lead to delayed healing, pathological scarring, or chronic non-healing wounds, which represent major clinical challenges in reconstructive and plastic surgery, particularly when compounded by systemic factors such as aging, metabolic disorders, or genetic predisposition [5,6,7,8,9].

Despite considerable progress in operative techniques, perioperative management, and biomaterial innovation, postoperative complications continue to represent a major clinical concern [10,11]. On the other hand, the global demand for reconstructive and plastic interventions continues to expand for the restoration of anatomical integrity, functional capacity, and overall quality of life in patients treated for congenital malformations, traumatic injuries, oncologic defects, degenerative disorders, or aesthetic concerns [12,13,14].

The initial inflammatory phase is orchestrated by recruitment of immune cells and production of pro-inflammatory mediators, including interleukin-6 (IL-6), which drives leukocyte activation and propagation of the repair cascade [7,15,16]. As healing progresses into the proliferative phase, fibroblasts and myofibroblasts synthesize ECM proteins such as type III and type I collagens, which form the structural scaffold of granulation tissue and ultimately a mature scar [6,17]. The balance between collagen synthesis and degradation is further modulated by matrix metalloproteinases (e.g., MMP9), enzymes essential for remodelling ECM and enabling cellular migration and wound closure [2,18,19].

Genetic factors influence nearly every phase of wound repair. Monogenic disorders affecting connective tissue and ECM proteins highlight the critical role of gene function in repair fidelity, while polygenic influences modulate susceptibility to impaired healing in common conditions such as diabetes and aging [6,8,16,20,21,22]. Beyond structural genes, polymorphisms and differential expression of genes involved in inflammatory signalling (e.g., interleukin-6), stress signalling pathways (Mitogen-Activated Protein Kinase 1/8—MAPK1/MAPK8), and ECM remodelling (metalloproteinase-9—MMP9, structural extracellular matrix proteins—Collagen type I (III) alpha 1 chain—COL1A1/COL3A1, and transforming growth factor-β1—TGFB1) have been implicated in inter-individual variation in healing outcomes and risk of adverse postoperative events [2,23,24,25,26].

Transforming growth factor-β1 is a central regulator of fibrogenesis and ECM deposition, integrating inflammatory cues with fibroblast activation and collagen production [27]. Dysregulated TGF-β1 signalling promotes excessive scar formation and fibrosis, a phenomenon frequently encountered in surgical patients with adverse postoperative trajectories [3,5,6,7]. Despite the recognized importance of these molecular pathways, the composite genetic modulation of inflammatory signalling, collagen synthesis, fibrogenesis, and matrix remodelling and their combined effect on postoperative complications remains incompletely characterized in cohorts undergoing reconstructive and plastic surgical procedures.

The selection of candidate genes in the present study was based on a hypothesis-driven approach grounded in the established molecular biology of wound healing. Specifically, we focused on genes representing key, functionally distinct phases of tissue repair. Pro-inflammatory mediators such as IL6 and the chemokine CCL2 were included due to their central role in leukocyte recruitment, cytokine signalling, and initiation of the inflammatory cascade [3,6,7,15]. Stress-activated kinases MAPK1 and MAPK8 were selected as critical regulators of cellular responses to hypoxia, oxidative stress, and apoptosis, which are known to influence tissue regeneration and repair dynamics [2,23].

In addition, genes involved in extracellular matrix (ECM) turnover and remodelling were incorporated, including MMP9, which mediates matrix degradation and facilitates cellular migration, and structural proteins COL1A1 and COL3A1, which are essential for collagen deposition and scar formation [18,19]. The cytokine TGFB1 was included as a master regulator of fibrogenesis, coordinating fibroblast activation, collagen synthesis, and tissue remodelling [26,27]. The anti-inflammatory cytokine IL10 was analysed as a key modulator of immune response resolution and inflammatory balance.

These genes have been consistently implicated in impaired wound healing, fibrosis, and postoperative complications in both experimental and clinical studies [20]. Therefore, their combined assessment was intended to capture the integrated molecular signature of inflammation, stress signalling, and fibrogenesis that underlies variability in postoperative outcomes. Importantly, gene selection was predefined based on biological plausibility and prior evidence, rather than derived from exploratory high-throughput screening, thereby strengthening the mechanistic rationale of the study. Understanding how genetic expression profiles of key wound healing mediators relate to clinical outcomes can provide a rationale for personalized risk stratification and targeted therapeutic interventions. The combined effect of multiple wound-healing-related genes on postoperative complications in plastic surgery remains insufficiently characterized.

Therefore, the objective of our research was to investigate the patterns of mRNA expression of critical genes including IL-6, CCL2, IL-10, MAPK1, MAPK8, MMP9, COL1A1, COL3A1 and TGFB1 and their associations with adverse postoperative outcomes in reconstructive and plastic surgery patients, depending on age.

## 2. Materials and Methods

### 2.1. Compliance with Bioethics

This study was conducted in full compliance with the European Convention on Human Rights and Biomedicine, the principles of Good Clinical Practice (GCP) and Good Laboratory Practice (GLP), EU Council Directive No. 609, and other relevant European and international bioethical regulations. The study protocol and patient consent statement were approved by the Ethics Committee of the Bukovinian State Medical University (Protocol No. 1 from 19 September 2024). This study is defined as a prospective longitudinal cohort study. Patients informed consent was signed by every participating individual.

### 2.2. Clinical Material

Clinical data were collected at the “Bukovinian Centre of Plastic and Aesthetic Surgery” (Chernivtsi, Ukraine) during 2024 and 2025.

Inclusion criteria included adult healthy women undergoing elective reconstructive or aesthetic breast surgery, age 19 and older, absence of acute inflammatory disease, and provision of informed consent.

Exclusion criteria included active infection, severe systemic diseases, autoimmune disease, immunosuppressive therapy, malignancy recurrence, and incomplete clinical data, and communication barriers preventing reliable data collection (mostly due to psychiatric or severe psychological disorders).

A total of 95 apparently healthy women were enrolled in this prospective longitudinal cohort study and underwent reconstructive or plastic breast surgery. The mean age was 35.48 ± 6.61 years (range: 19–57 years). All participants provided written informed consent prior to inclusion and underwent comprehensive demographic, clinical, instrumental (breast ultrasound or mammography), and laboratory evaluations, including complete blood count and biochemical testing.

### 2.3. Groups Stratification

Participants were stratified by age (<30 years—24.21%; 30–39 years—44.21%; ≥40 years—31.58%), presence of postoperative complications (complication rate—12.63%; non-complicated—87.67%), and type of surgical procedure: augmentation mammoplasty (69.47%), reduction mammoplasty (5.26%), mastopexy (6.32%), mastopexy combined with mammoplasty (9.47%), implant replacement (7.37%), and implant removal (2.11%).

Documented postoperative complications were categorized into short-term such as retromammary seroma (2.11% cases) and long-term events (capsular fibrosis (6.32%), implant malposition with breast contour deformation (3.16%), and implant rupture (1.05%) with standardized diagnostic criteria. Short-term complications, occurring in the perioperative period, may compromise reconstructive success. In contrast, long-term complications generally do not pose an immediate risk of reconstruction failure but involve unfavourable aesthetic outcomes.

Documented postoperative complications in anamnesis included retromammary seroma (2.11%), capsular fibrosis (6.32%), implant malposition with breast contour deformation (3.16%), and implant rupture (1.05%). A history of previous surgical interventions in anamnesis at other anatomical sites was reported by 24.21% of participants (such as tonsillectomy, appendectomy, Caesarean section, or face lift surgery), while 14.74% had concomitant chronic conditions, including cystitis, pyelonephritis, Gilbert’s syndrome, bronchitis, or meniscal injury.

All women operated on were non-smokers. Body mass index (BMI) ranged from 17.58 to 25.71 kg/m^2^ (mean 21.13 ± 1.98 kg/m^2^).

### 2.4. Transcriptional Activity of IL6, CCL2, MAPK1, MAPK8, IL10, MMP9, COL1A1 and COL3A1 Genes

**Figure 1:**
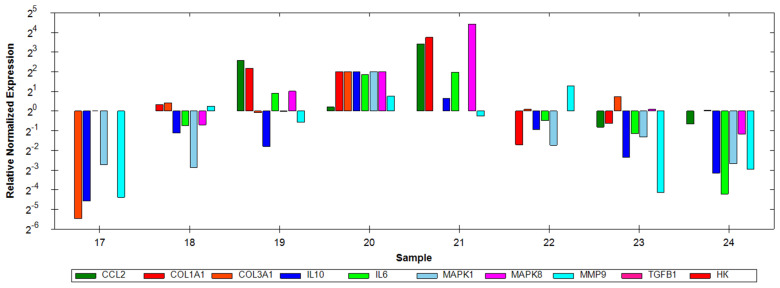
Relative normalized expression of the IL-6, CCL2, MAPK1, MAPK8, IL-10, MMP9, COL1A1, COL3A1, and TGFβ1 genes in the blood with the reference gene GAPDH (−2·2 LogN, ΔΔCt).

Transcriptional activity of IL6, CCL2, MAPK1, MAPK8, IL10, MMP9, COL1A1 and COL3A1 genes in the peripheral blood leukocytes was assessed using a PCR with iTaq Universal SYBR Green Supermix (SMX500; Thermo Fisher Scientific Inc., Bio-Rad Laboratories, Waltham, MA, USA). Total RNA was isolated using the GeneAll^®^ Hybrid-R™ Blood RNA Kit (GeneAll Biotechnology, Seoul, Republic of Korea) according to the manufacturer’s instructions and reverse-transcribed into cDNA using the iScript™ gDNA Clear cDNA Synthesis Kit (Bio-Rad Laboratories, USA). RNA samples within the −2.0 to 2.2 range were included, and all reactions were performed in triplicate. Relative gene expression levels were quantified using glyceraldehyde-3-phosphate dehydrogenase (GAPDH) as the reference gene and normalized using the ΔΔCt method (Figure 1). Fold changes in gene expression were calculated using the 2^−ΔΔCt^ formula. The transcriptional activity assessment protocol was described in our former publications [28,29]. Genetic examination was performed for 50 women/participants. The licensed CFX96 RT-PCR Detection System Software (Bio-Rad CFX Maestro Software, Bio-Rad Laboratories, USA), version 4.0.2325.0418, was used for data acquisition and analysis (Figure 1).

### 2.5. Statistical Analysis of PCR Array Data

The RT^2^ Profiler PCR Array Data Analysis software (QIAGEN, Hilden, Germany; accessed on January, 2026) provides limited statistical evaluation, restricted to the calculation of *p*-values using a two-tailed Student’s t-test (assuming equal variances) based on triplicate 2^−ΔCt^ values of the IL6, CCL2, MAPK1, MAPK8, IL10, MMP9, COL1A1, and COL3A1 genes in patients compared with controls. Microarray Quality Control assessment confirmed that the sample size and statistical approach were adequate to ensure reproducibility across microarray platforms and PCR arrays, including the RT^2^ Profiler PCR Arrays.

### 2.6. Statistical Analysis

Statistical analyses were performed using Statistica 7.0 (StatSoft Inc., Tulsa, OK, USA) and Microsoft Excel^®^ 2016 software packages. Categorical variables were analysed using odds ratios (ORs) with 95% confidence intervals (CIs) and Pearson’s chi-square test (χ^2^) (df = 1); Fisher’s exact test was applied when expected frequencies were <5. Multivariate logistic regression analysis was performed to assess independent predictors by relative risk (RelR), risk ratio (RR), OR with 95% CI and χ^2^ (df = 1). Continuous variables were analysed using Student’s t test (two-tailed, assuming equal variances), or the Wilcoxon-Mann–Whitney U test for non-normally distributed data, as determined by the Shapiro–Wilk or Kolmogorov–Smirnov tests. Data are presented as mean (M) or median (Me) with interquartile range (Q1–Q3).

*Multiple testing clarification.*

Given the number of genes analysed, the issue of multiple comparisons was considered. Due to the exploratory and hypothesis-driven nature of the study and the limited sample size, formal correction methods (e.g., Bonferroni adjustment) were not applied, as they may increase the risk of type II error in small datasets. Instead, the interpretation of results was based on the consistency of associations, effect sizes, and biological plausibility. Nonetheless, the potential for type I error inflation is acknowledged.

*Development of the Molecular Complication Risk Index (MCRI).*

The MCRI was developed to provide an integrated estimate of postoperative complication risk based on gene expression profiles. The index was constructed using a multivariable logistic regression framework, where postoperative complications (binary outcome) were modelled as a function of normalized gene expression levels (ΔΔCt values).

MCRI = *β*0 + *β*1·*XTGFB*1 + *β*2·*XCOL*1*A*1 + *β*3·*XMMP*9 + *β*4·*XIL*-6 + ⋯ + *βn*·*Xn*,

where *β*0 = intercept; *β*i = regression coefficient for each molecular marker; and Xi = normalized mRNA expression level of gene i.

To enhance clinical interpretability and reduce model instability associated with small sample size, gene expression values were categorized into ordinal levels based on cohort-specific quartiles (low: ≤Q1, moderate: Q1–Q3, and high: >Q3). Each marker was assigned a weight reflecting both the magnitude of its association with complications (based on regression coefficients and odds ratios) and biological relevance in wound healing pathways. MRCI was transformed into weights by a weighted linear score to analyse postoperative complication marker burden:

MRCI = ∑_(i = 1)^nω1·Zi,

where ωi = standardized regression weight and Zi = standardized (z-score normalized) gene expression value.

The final MCRI score was calculated as a weighted linear combination of standardized gene expression values. For practical application, the continuous score was additionally transformed into a simplified point-based system. It should be noted that the weighting scheme was partially informed by biological plausibility and observed effect sizes rather than derived from a fully data-driven optimization procedure, due to the limited sample size.

The MCRI was constructed using a multivariable logistic regression model, where the probability of postoperative complications (P) was modelled as a function of gene expression levels. The general form of the model was as follows:

logit(P) = β_0_ + β_1_·IL6 + β_2_·CCL2 + β_3_·MAPK1 + β_4_·MAPK8 + β_5_·IL10 + β_6_·MMP9 + β_7_·COL1A1 + β_8_·COL3A1 (+ β_9_·TGFB1, if applicable),

where β coefficients represent regression weights estimated from the data.

To improve interpretability and reduce the influence of outliers, gene expression values were standardised and, where appropriate, categorised into ordinal levels based on cohort-specific thresholds. The weighting scheme for the final index was primarily based on the magnitude of regression coefficients, with consideration of biological relevance in wound healing pathways. The resulting MCRI score was calculated as a weighted sum of predictor variables.

Statistical significance was defined as *p* < 0.05.

## 3. Results

### 3.1. Comparative Gene Expression Analysis According to Postoperative Outcomes

Comparative analysis of normalized gene expression levels (ΔΔCt, median [Q1–Q3]; mean) between patients with and without postoperative complications revealed a statistically significant upregulation of most investigated markers in the complication group (Table 1). Given the asymmetric distribution of values and the presence of zero-expression measurements, the Mann–Whitney U test (Z-score) was applied in addition to mean comparisons, which is methodologically justified for non-normally distributed molecular data.

In patients without complications, IL-6 and CCL2 expression levels were low (mean −0.55 and −1.66, respectively). In contrast, patients who developed postoperative complications demonstrated significant upregulation of IL-6 (median 1.62; mean 1.44; Z = −2.63; *p* = 0.004) and CCL2 (median 1.24; mean 1.31; Z = −3.69; *p* < 0.001).

### 3.2. Stress-Activated MAPK Signalling

Marked activation of MAPK1 and particularly MAPK8 was observed in the complication group, suggesting activation of stress-induced signalling cascades associated with tissue hypoxia, apoptosis, impaired regeneration, and delayed wound healing. MAPK1 and MAPK8 demonstrated some of the largest intergroup differences: MAPK1: mean −0.67 vs. 2.03 (*p* < 0.001), more than a three-fold difference; MAPK8: mean −0.35 vs. 2.91 (*p* < 0.001), approximately an eight-fold difference (Table 1).

### 3.3. Anti-Inflammatory Response and Matrix Degradation

Interestingly, IL-10 expression was significantly elevated in the complication group (mean 1.82; *p* < 0.001), nearly two-fold compared with the non-complication group. This likely reflects a compensatory immunoregulatory response secondary to excessive inflammation, although insufficient to restore inflammatory balance.

MMP9 expression was also significantly increased in patients with complications (mean 1.94; *p* < 0.001), nearly doubling relative to the group without complications (Table 1).

### 3.4. Fibrogenesis and Collagen Imbalance

A pronounced increase in COL1A1 expression was observed in the complication group (median 3.51; mean 3.47; *p* < 0.001), exceeding levels in the non-complication group by more than six-fold in logarithmic scale (median −0.56; mean −0.51). Concurrently, COL3A1 expression increased more than two-fold (mean 2.21; *p* = 0.001) (Table 1).

Although TGFB1 expression did not reach statistical significance, a trend toward higher expression was observed in patients with complications (median 2.66; mean 2.87), nearly four-fold higher than in patients without complications. This suggests activation of fibroproliferative mechanisms in response to impaired healing. However, isolated TGFB1 expression does not appear to function as an independent predictor; its clinical relevance likely depends on interaction with other inflammatory and ECM-remodelling genes.

### 3.5. Integrated Molecular Phenotype of Postoperative Complications

Overall, patients with postoperative complications demonstrated increased expression of pro-inflammatory and chemotactic genes (IL-6 and CCL2), overactivation of MAPK stress signalling pathways (MAPK1 and MAPK8), compensatory elevation of anti-inflammatory signalling (IL-10), dysregulated ECM remodelling (MMP9), and enhanced fibrogenesis (COL1A1, COL3A1, and TGFB1). This molecular profile reflects a high-risk inflammatory-fibroproliferative phenotype associated with unfavourable surgical outcomes.

### 3.6. Epidemiological Risk Analysis

Epidemiological modelling confirmed a significant increase in the probability of postoperative complications associated with elevated gene expression levels (Table 2): IL-6 (≥1.25) and COL3A1 (≥1.15), OR = 7.47 (95% CI 1.77–31.56; *p* = 0.007); CCL2 (≥1.56), OR = 6.2 (95% CI 1.51–25.41; *p* = 0.012); MAPK1 (≥1.12), OR = 25.5 (95% CI 4.81–135.13; *p* < 0.001); MAPK8 (≥1.71), IL-10 (≥0.99), and MMP9 (>1.28), OR = 13.2 (95% CI 2.87–60.65; *p* < 0.001); and COL1A1 (>2.31), OR = 58.33 (95% CI 8.53–398.81; *p* < 0.001). The strongest association was observed for COL1A1 overexpression.

### 3.7. Molecular Complication Risk Index (MCRI)

To provide an integrated assessment of molecular risk, a Molecular Complication Risk Index (MCRI) was developed based on a weighted sum of normalized gene expression levels (ΔΔCt) involved in inflammation, stress signalling, and ECM remodelling. The MCRI score was derived directly from the regression model coefficients and reflects the combined contribution of individual gene expression markers. Given ΔΔCt-based expression values, rank normalization was applied: low expression (Me ≤ Q1) = 0 points, moderate expression (Q1 < Me ≤ Q3) = 1 point, and high expression (Me > Q3) = 2 points. MAPK1 was assigned a negative weight, as higher levels may reflect preserved reparative potential. Marker weights are presented in the corresponding Table 3.

**Figure 2:**
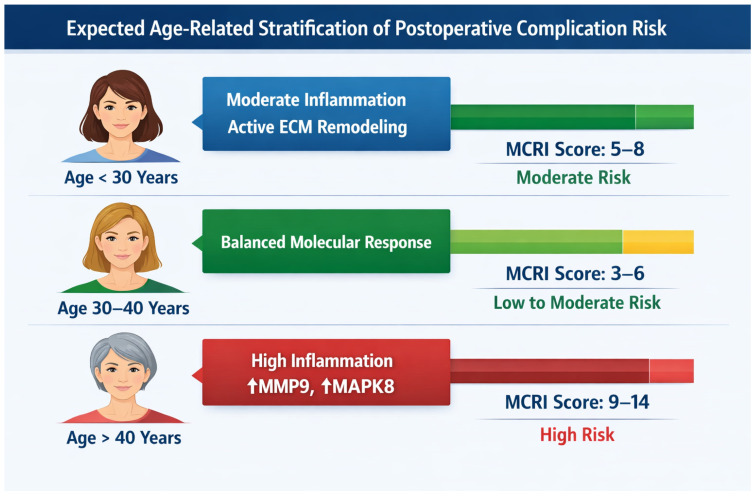
Image of age-related stratification of postoperative complication risk based on MCRI.

The expected age-related stratification of postoperative complication risk is presented in Figure 2. According to the MCRI score, 0–5 points indicate a low risk of postoperative complications (physiological wound healing), 6–10 points correspond to a moderate risk (seroma formation, prolonged inflammation), and MCRI ≥ 11 points indicate a high risk (infection, necrosis, fibrosis, capsular contracture). The analysis demonstrates that the highest MCRI values, reflecting an unfavourable molecular profile, are predominantly associated with women older than 40 years.

**Figure 3:**
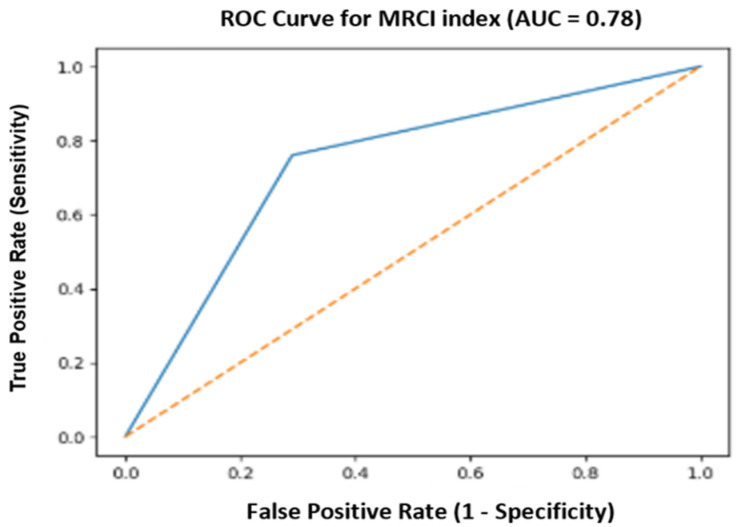
ROC curve of the Molecular Complication Risk Index (MCRI) for predicting postoperative complications.

ROC curve analysis demonstrated good (moderately high) discriminatory performance of MCRI for predicting postoperative complications, with AUC = 0.78 (95% CI 0.68–0.87) and Youden index = 0.47 (*p* < 0.05) (Figure 3). The optimal ROC-derived cut-off value (≥8.5 points) yielded sensitivity = 76% and specificity = 71%.

**Figure 4:**
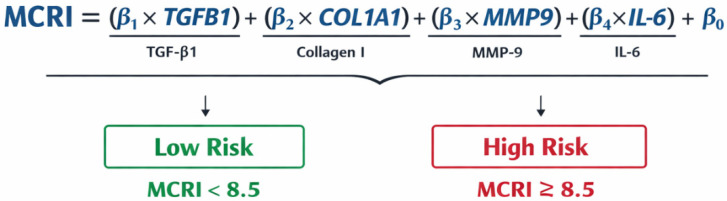
Risk of adverse postoperative outcomes based on MCRI.

The equation and MRCI risk score are presented in Figure 4. Patients were classified as having a high risk of adverse postoperative outcomes when MCRI ≥ 8.5. Higher MCRI values (≥8.5) were significantly associated with increased risk. The MCRI demonstrates adequate performance for clinical risk screening, though not for standalone diagnostic decision-making. The most informative threshold corresponds to the transition from moderate to high molecular risk. Given that model development and evaluation were conducted within the same dataset, the reported performance should be interpreted with consideration of potential optimism.

### 3.8. The Impact of Surgical Complexity and BMI on the Risk of Postoperative Complication

None of the women were smokers, therefore, smoking as a possible risk factor of postoperative complication was excluded. BMI increased with the age of the women (under 30 years—21.14 ± 2.08 kg/m^2^; 30–40 years—22.65 ± 1.71 kg/m^2^ (*p* = 0.04); over 40 years—22.09 ± 1.95 kg/m^2^) and was lower in women with postoperative complications (20.75 ± 1.50 kg/m^2^ versus 22.56 ± 1.86 kg/m^2^; *p* = 0.01). The complexity of surgical intervention was associated with the frequency of complications (r = 0.65; *p* = 0.035). The probability of complications increased with the complexity of surgical interventions by more than nine times (OR = 9.07; OR95%CI: 2.41–8.08; *p* = 0.001) and with a low BMI (≤22 kg/m^2^) by almost 12 times (OR = 11.64; OR95%CI: 4.59–13.93; *p* < 0.001).

## 4. Discussion

The present cohort study demonstrates that adverse postoperative outcomes in reconstructive and plastic surgery are associated with a distinct molecular pattern characterized by coordinated upregulation of inflammatory mediators, stress-activated signalling pathways, extracellular matrix (ECM) remodelling enzymes, and fibrogenic genes. Collectively, these alterations define a high-risk inflammatory—fibroproliferative phenotype that may underlie impaired wound healing and pathological scar formation.

Persistent inflammatory activation is a driver of complications. We observed significant upregulation of IL-6 and CCL2 in patients who developed postoperative complications. IL-6 is a central mediator of acute-phase inflammatory responses and plays a dual role in wound repair: while transient expression supports host defence and tissue regeneration, sustained elevation promotes chronic inflammation and fibrosis [7,30,31,32]. Similarly, CCL2 (MCP-1) orchestrates monocyte/macrophage recruitment, influencing the inflammatory microenvironment and subsequent fibroblast activation [33,34,35]. Excessive or prolonged chemotactic signalling may predispose to seroma formation, surgical site infection, and delayed wound resolution [36]. These findings indicate persistent activation of pro-inflammatory mediators, enhanced monocyte recruitment, and intensified chemotaxis—mechanisms critically involved in seroma formation, infection, and chronic postoperative inflammation. Moreover, our findings support the concept that dysregulated inflammatory persistence, rather than insufficient activation, contributes to unfavourable postoperative trajectories.

Importantly, the concurrent increase in IL-10 expression in the complication group likely reflects a compensatory immunoregulatory response. IL-10 suppresses pro-inflammatory cytokine production and modulates macrophage polarization [37,38,39]. However, compensatory anti-inflammatory signalling may be insufficient to counterbalance excessive IL-6/CCL2-driven responses, leading to an unresolved inflammatory milieu that interferes with orderly repair.

MAPK pathway activation and stress signalling. Among the most pronounced differences between groups were MAPK1 (Extracellular Signal-Regulated Kinase 2—ERK2) and MAPK8 (JNK1). These kinases integrate signals from inflammatory cytokines, oxidative stress, and hypoxia, regulating proliferation, apoptosis, and fibroblast activation [40,41]. The marked upregulation of MAPK8 in particular suggests activation of stress-responsive cascades associated with tissue hypoxia and prolonged wound healing. JNK activation has been implicated in fibroblast differentiation and excessive ECM deposition under pathological conditions [42,43].

Interestingly, while MAPK1 activation is typically associated with proliferative signalling and tissue repair, excessive or sustained ERK pathway activation may promote fibrosis and aberrant remodelling [43,44,45,46,47]. These findings demonstrate strong activation of proliferative and stress-dependent pathways and highlight their potential as predictors of adverse postoperative outcomes. The magnitude of MAPK1- and MAPK8-associated odds ratios in our epidemiological analysis underscores their potential utility as predictive biomarkers in surgical risk stratification.

Extracellular matrix remodelling imbalance is one of the key factors in wound healing. Successful wound healing requires tightly regulated ECM degradation and synthesis. Our results demonstrated significant upregulation of MMP9 in the group with postoperative complications, indicating enhanced matrix degradation. This finding suggests excessive ECM degradation, which clinically may manifest as impaired wound stability, seroma formation, delayed maturation of scar tissue, or chronic wound healing disturbances. MMP9 facilitates leukocyte migration and early matrix turnover; however, persistent elevation may destabilize granulation tissue and delay maturation of the wound matrix [2,48]. Excessive proteolytic activity has been linked to chronic wounds and impaired epithelialization [49].

Simultaneously, COL1A1 and COL3A1 expression was markedly increased in patients with complications, reflecting enhanced fibrogenesis. Type III collagen predominates during early repair, whereas type I collagen becomes dominant in later remodelling phases. Disruption of the balance between these collagen types may result in disorganized matrix architecture, fibrosis, capsular contracture, or hypertrophic scar formation [50,51]. The particularly strong association between COL1A1 overexpression and complication risk in our cohort highlights the central role of exaggerated fibrotic signalling in adverse surgical outcomes. These findings indicate disorganized fibrogenesis characterized by imbalance between early (type III) and late (type I) collagen deposition, forming the molecular basis of fibrosis, capsular contracture, and pathological scarring.

TGF-β1 is integrated fibroproliferative signalling. Although TGFB1 did not reach statistical significance, its trend toward increased expression in complicated cases aligns with its established role as a master regulator of fibrogenesis. TGF-β1 stimulates fibroblast proliferation, myofibroblasts differentiation, and collagen synthesis while inhibiting matrix degradation [27,52,53]. It also interacts with MAPK and Smad-dependent pathways, amplifying fibrotic responses [53,54,55]. Our findings suggest that TGFB1 may exert synergistic rather than independent effects, reinforcing the concept that postoperative complications arise from network-level dysregulation rather than single-gene perturbations.

Integrated molecular risk profiling. To translate these findings into clinical practice, we developed a Molecular Complication Risk Index (MCRI) integrating expression levels of key inflammatory, stress-signalling, and ECM-remodelling genes. ROC analysis demonstrated good discriminative performance (AUC 0.78), supporting the feasibility of molecular risk stratification. While not suitable as a standalone diagnostic tool, MCRI may complement clinical and surgical risk factors in preoperative assessment. On the other hand, the proposed MCRI demonstrates moderate discriminatory ability; however, it requires further validation before it can be applied in routine clinical practice.

Personalized risk profiling based on gene expression has been increasingly proposed in regenerative medicine and surgical sciences [20]. Identification of high-risk molecular phenotypes could inform perioperative modulation strategies, including anti-inflammatory optimization, targeted anti-fibrotic therapies, or enhanced postoperative monitoring.

Clinical implications. The identified molecular pattern suggests that adverse postoperative outcomes are not merely the result of technical or local factors but reflect systemic and genetic predispositions influencing inflammatory intensity and fibrotic potential. Preoperative identification of patients with exaggerated inflammatory-fibrogenic responses may enable tailored surgical planning, individualized perioperative anti-inflammatory or anti-fibrotic interventions, and intensified postoperative surveillance. These findings align with emerging paradigms of precision surgery and molecularly informed risk prediction.

Clinical confounders. The present findings indicate that BMI tends to increase with age. Notably, lower BMI (≤22 kg/m^2^) was significantly associated with the occurrence of postoperative complications—an almost twelve-fold increase in complication risk. This finding highlights the potential role of body composition as an independent risk factor, possibly mediated through decreased subcutaneous tissue support, impaired vascularization, and altered inflammatory/immune responses. Moreover, our observation suggests that insufficient body mass may reflect reduced metabolic reserves and impaired nutritional status, which can negatively affect wound healing and tissue remodelling.

Importantly, the risk of complications was strongly influenced by both patient-related and procedure-related factors. Surgical complexity emerged as a major determinant, increasing the likelihood of complications more than nine-fold. This is consistent with the greater extent of tissue dissection, longer operative time, and higher risk of intraoperative trauma associated with more complex procedures.

### Limitations

This study is limited by cohort size and single-time-point gene expression assessment. Therefore, the effect sizes and large ORs, should be interpreted cautiously considering the limited sample size and corresponding confidence intervals. Dynamic longitudinal profiling could provide additional insight into temporal regulation of inflammatory and fibrotic pathways. Furthermore, protein-level validation and mechanistic studies are warranted to confirm causal relationships. Moreover, the study is exploratory and hypothesis-generating; the sample size reflects a single-centre cohort with strict inclusion criteria; and the restriction to female patients reflects the clinical specificity of breast surgery populations, but limits generalisability. Future studies involving larger, multi-centre, and more diverse cohorts may help to further validate these findings.

Several limitations related to the MCRI model should be acknowledged. First, the index was developed and tested within the same cohort without internal (e.g., cross-validation or bootstrapping) or external validation, which may result in overestimation of predictive performance. Second, the relatively small sample size and limited number of complication events increase the risk of model overfitting and reduce the stability of regression coefficients. Third, the weighting of individual variables was partially based on biological plausibility in addition to statistical associations, which, while mechanistically justified, introduces a degree of subjectivity. Therefore, the MCRI should be considered a preliminary, hypothesis-generating tool that requires validation in larger, independent, and multi-centre cohorts before clinical implementation.

Although internal validation procedures such as cross-validation or bootstrapping were not performed in the present study due to the limited sample size, it is likely that the reported model performance (AUC) is subject to optimism bias. In similar settings, internal validation typically results in some attenuation of predictive performance. Therefore, the current estimates should be interpreted as preliminary, and future studies should include appropriate validation strategies to assess model stability, calibration, and generalisability.

Peripheral blood gene expression reflects systemic inflammatory and immune response. The relationship between systemic transcriptional activity and local wound healing requires further validation, including tissue-level studies.

## 5. Conclusions

In this combined retrospective and prospective study, specific gene expression patterns related to inflammation, stress signalling, and extracellular matrix remodelling were associated with an increased risk of postoperative complications following breast plastic and reconstructive surgery.

The proposed Molecular Complication Risk Index (MCRI) demonstrated moderate discriminatory ability in this cohort and may represent a promising approach for molecular risk stratification. However, given the observational design, limited sample size, and lack of external validation, further large-scale, multi-centre studies are required to validate these results and to determine the clinical utility of incorporating molecular markers into perioperative risk assessment and personalised surgical strategies.

Lower body mass index (BMI) and increased surgical complexity are independent predictors of postoperative complications, underscoring the importance of preoperative risk stratification and individualized surgical planning.

## Figures and Tables

**Table 1:** Genetic molecular marker panel of postoperative complications according to normalized gene expression and complication status.

Marker	Gene Expression mRNA Me (Q1; Q3); M		
	Women Without Complications	Women with Complications	(U_Me_) Z-Score; *p* (p_M_)
IL-6	0 (−0.67; 0.82); M: −0.55	1.62 (−1.49; 2.07) M: 1.44	Z = −2.63; *p* = 0.004 (p_M_ = 0.032)
CCL2	0 (−2.75; 0.56) M: −1.66	1.24 (−0.46; 2.47) M: 1.31	Z = −3.69; *p* < 0.001 (p_M_ = 0.031)
MAPK1	0 (−1.73; 0.45) M: −0.67	1.61 (1.0; 2.15) M: 2.03	Z = −3.99; *p* < 0.001 (p_M_ < 0.001)
MAPK8	−0.45 (−1.30; 1.04) M: −0.35	2.70 (1.02; 3.49) M: 2.91	Z = −3.0; *p* = 0.001 (p_M_ < 0.001)
IL-10	−0.79 (−2.95; 0.02) M: −1.28	2.21 (0.90; 2.58) M: 1.82	Z = −1.71; *p* = 0.043 (p_M_ < 0.001)
MMP9	−0.78 (−2.96; 0.41) M: −1.53	1.51 (0.86; 2.59) M: 1.94	Z = −1.86; *p* = 0.031 (p_M_ < 0.001)
COL1A1	−0.56 (−1.71; 0.42) M: −0.51	3.51 (2.32; 4.26) M: 3.47	Z = −2.16; *p* = 0.015 (p_M_ < 0.001)
COL3A1	0.03 (−0.58; 0.54) M: −0.39	1.58 (0.57; 2.30) M: 2.21	Z = −2.88; *p* = 0.002 (p_M_ = 0.001)
TGFβ1	0.80 (−1.10; 2.58) M: 0.72	2.66 (1.75; 3.57) M: 2.87	Z = −0.84; *p* = 0.201 (p_M_ = 0.185)

**Table 2:** Risk of postoperative complications based on a genetic-molecular panel of transcriptional gene activity.

Markers (≥Q3 LogN)	RR	RR 95%CI	OR	OR 95%CI	*p*
IL-6 (≥1.25)	3.98	1.53–10.39	7.47	1.77–31.56	0.007
CCL2 (≥1.56)	3.60	1.37–3.47	6.20	1.51–25.41	0.012
MAPK1 (≥1.12)	8.54	2.72–26.80	25.50	4.81–135.13	<0.001
MAPK8 (≥1.71)	5.69	2.05–15.79	13.20	2.87–60.65	<0.001
IL-10 (≥0.99)	5.69	2.05–15.79	13.20	2.87–60.65	<0.001
MMP9 (>1.28)	5.69	2.05–15.79	13.20	2.87–60.65	<0.001
COL1A1 (>2.31)	14.23	3.58–56.59	58.33	8.53–398.81	<0.001
COL3A1 (≥1.15)	3.98	1.53–10.39	7.47	1.77–31.56	0.007
TGFB1 (≥2.58)	6.33	0.63–63.87	7.40	0.61–90.16	0.139

**Table 3:** Weight coefficients of the analysed postoperative complication markers.

Marker	Weight Coefficient	Rationale, Explanation
IL-6	2.0	Key pro-inflammatory mediator
CCL2	1.5	Macrophage infiltration
MAPK8	2.0	Stress, apoptosis, ischemia
IL-10	1.0	Compensatory immunosuppression, anti-inflammatory mediator
MMP9	2.0	Seroma, degradation of extracellular matrix
COL1A1	1.5	Fibrosis, scarring
COL3A1	1.0	Scar remodelling
MAPK1	−0.5	Adaptive-proliferative factor

## Data Availability

Data are available on request from the corresponding authors.

## Abbreviations

The following abbreviations are used in this manuscript:

COL1A1	collagen type I alpha 1 chain
COL3A1	collagen type III alpha 1 chain
TGFB1	transforming growth factor-β1
CCL2	chemokines including C-C motif chemokine ligand 2
GCP	Good Clinical Practice
GLP	Good Laboratory Practice
GAPDH	glyceraldehyde-3-phosphate dehydrogenase
MCRI	molecular complication risk index
ERK2	extracellular signal-regulated kinase 2
